# Mixed methods feasibility study for a trial of blood pressure telemonitoring for people who have had stroke/transient ischaemic attack (TIA)

**DOI:** 10.1186/s13063-015-0628-y

**Published:** 2015-03-25

**Authors:** Janet Hanley, Peter Fairbrother, Ashma Krishan, Lucy McCloughan, Paul Padfield, Mary Paterson, Hilary Pinnock, Aziz Sheikh, Cathie Sudlow, Allison Todd, Brian McKinstry

**Affiliations:** Edinburgh Napier University, Sighthill Campus, Sighthill Court, Edinburgh, EH11 4BN UK; Edinburgh Health Services Research Unit/Edinburgh Clinical Trials Unit, Western General Hospital, Crewe Road, Edinburgh, EH4 2XU UK; Telescot Programme, Centre for Population Health Sciences, The University of Edinburgh, Teviot Place, Edinburgh, EH8 9DX UK; NHS Lothian, C/O Telescot Programme, Centre for Population Health Sciences, The University of Edinburgh, Teviot Place, Edinburgh, EH8 9DX UK

**Keywords:** Hypertension, Stroke, Transient Ischaemic Attack, General Practice, Home monitoring, Telemonitoring, Pilot Randomised Controlled Trial, Qualitative Research

## Abstract

**Background:**

Good blood pressure (BP) control reduces the risk of recurrence of stroke/transient ischaemic attack (TIA). Although there is strong evidence that BP telemonitoring helps achieve good control, none of the major trials have considered the effectiveness in stroke/TIA survivors. We therefore conducted a feasibility study for a trial of BP telemonitoring for stroke/TIA survivors with uncontrolled BP in primary care.

**Method:**

Phase 1 was a pilot trial involving 55 patients stratified by stroke/TIA randomised 3:1 to BP telemonitoring for 6 months or usual care. Phase 2 was a qualitative evaluation and comprised semi-structured interviews with 16 trial participants who received telemonitoring and 3 focus groups with 23 members of stroke support groups and 7 carers.

**Results:**

Overall, 125 patients (60 stroke patients, 65 TIA patients) were approached and 55 (44%) patients were randomised including 27 stroke patients and 28 TIA patients. Fifty-two participants (95%) attended the 6-month follow-up appointment, but one declined the second daytime ambulatory blood pressure monitoring (ABPM) measurement resulting in a 93% completion rate for ABPM − the proposed primary outcome measure for a full trial. Adherence to telemonitoring was good; of the 40 participants who were telemonitoring, 38 continued to provide readings throughout the 6 months. There was a mean reduction of 10.1 mmHg in systolic ABPM in the telemonitoring group compared with 3.8 mmHg in the control group, which suggested the potential for a substantial effect from telemonitoring. Our qualitative analysis found that many stroke patients were concerned about their BP and telemonitoring increased their engagement, was easy, convenient and reassuring.

**Conclusions:**

A full-scale trial is feasible, likely to recruit well and have good rates of compliance and follow-up.

**Trial Registration:**

ISRCTN61528726 15/12/2011.

**Electronic supplementary material:**

The online version of this article (doi:10.1186/s13063-015-0628-y) contains supplementary material, which is available to authorized users.

## Background

Ageing populations present a major challenge for health service planners worldwide. Policymakers in many countries see telehealth as a core component of plans to re-organise the management of long-term conditions [1,2], but it is not clear for which types of condition or for which patients this approach is likely to be most beneficial. In areas such as heart failure and chronic obstructive pulmonary disease, initial optimism surrounding telemonitoring – an application of telehealth where patients take measurements at home with a telemetry link to a healthcare provider – has been tempered by the more circumspect results from high-quality randomised controlled trials [3,4] and by the challenges of moving these interventions on from small demonstrator projects to normalise them into routine care at scale [5,6].

Stroke is the second most common cause of death in the world after ischaemic heart disease, causing around 9% of all deaths; it is also the commonest cause of adult disability in developed countries [7,8]. Raised blood pressure (BP) is the most important reversible risk factor for first or recurrent stroke, with risk increasing by about one-third for every 10 mmHg increase in systolic BP [9]. Patients with a previous stroke or transient ischaemic attack (TIA) have a particularly high risk of subsequent stroke, which is mitigated by long-term reduction in BP [10,11]. The greater the reduction in BP is, the greater the benefit, at least down to about 130/70 mmHg; few however achieve this challenging target [12,13].

There is strong evidence from systematic reviews [14,15] and recent randomised controlled trials [16,17] that telemonitoring is an effective way to achieve BP reduction in a general hypertensive population. However, telemonitoring is a complex intervention, sensitive to the context in which it is applied [18]. It cannot be assumed that the benefits seen in the general hypertensive population will necessarily apply in people with stroke or TIA (even though they have more to gain from good BP control), as they may be more debilitated [19,20] with more complex polypharmacy than the populations previously studied. Furthermore, clinicians may be cautious about lowering BP in these often elderly patients and, while only a minority of stroke and TIA survivors have severe residual disability [19], minor physical or cognitive disability may reduce the ability to use a BP monitoring system. Although there is evidence that some stroke and TIA survivors are willing and able to self-monitor their BP without telemonitoring [21,22], it did not improve BP control compared to a control group, in concordance with the limited impact of unsupported home BP monitoring found in trials in the general population [23,24]. Stroke/TIA survivors have not been studied as an a priori sub-group in previous telemonitoring trials [14-17], and poorer (or better) outcomes in stroke survivors may therefore have been masked. We aimed to test the feasibility of mounting a pragmatic randomised controlled trial of BP telemonitoring in patients who had a previous stroke/TIA in a UK primary care setting, using a protocol that had been effective in our previous trial [17] but that excluded this patient group. Our feasibility study aimed to investigate the likely recruitment rate to a trial, feasibility of using the telemonitoring service and the experiences and perspectives of those using the telemonitoring service and those who may not choose to do so.

## Methods

There were two major components to our mixed-methods evaluation. Phase 1 was a pilot trial involving stroke/TIA patients randomised in a ratio of 3:1 to telemonitoring or usual care. Phase 2 comprised interviews with pilot trial patients and nurses as well as focus groups with patients and carers attending stroke support groups. The study was approved by the South East Scotland Research Ethics Service (reference 11/SS/0023).

### Phase 1: Pilot trial

**Figure 1:**
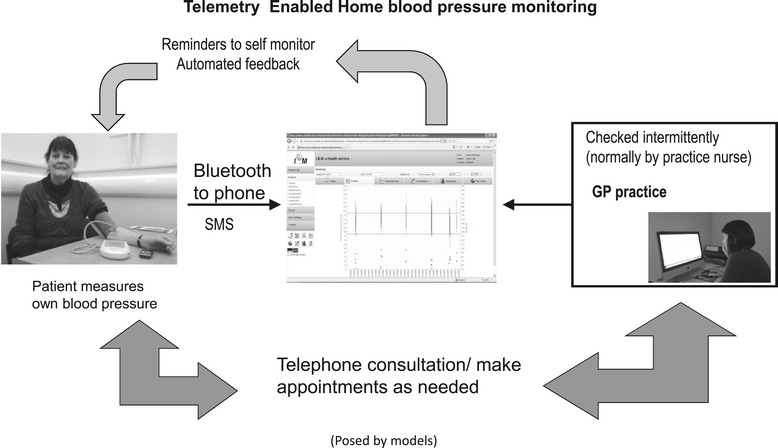
**The intervention.**

We undertook a pilot researcher-blinded randomised controlled trial in six socio-economically diverse general practices in Lothian, South East Scotland, to test the feasibility of the trial processes and measures. We included patients over 18 years of age with a history of stroke or TIA, whose most recent office-based BP measurement and subsequent daytime average ambulatory systolic BPs both exceeded 130 mmHg. Patients were excluded if they had: secondary hypertension; hypertension being managed in secondary care; a surgery measured BP of ≤120/60 mmHg or ≥220 mmHg systolic at the baseline research visit; terminal illness or major concurrent illness where treatment was likely to affect the ability to self-monitor; stroke within the last 3 months; major surgery within the last 3 months; atrial fibrillation (which would cause error messages from the automatic oscillometric monitors used); or inability to consent or use self-monitoring equipment alone and with no easy access to help. Details of the telemonitoring service are shown in Figure 1.

GPs from participating practices identified potentially eligible patients from practice computer records with the assistance of the Scottish Primary Care Research Network and outlined the study in a letter inviting them to express an interest via a reply slip. For stroke patients this correspondence included the patient information letter in regular format and stroke-accessible format. Stroke patients known to have disabilities that limited their ability to cope with written information were also telephoned by a member of the practice staff if necessary to ensure that they had received the information in an accessible format and that they were able to respond and return the reply slip if interested. Patients expressing an interest were contacted by the research nurse who arranged an appointment to see them at the practice (or at home if necessary).

The research nurse explained the intervention, demonstrated the equipment and gave the patient the opportunity to consider whether they would be able to use the equipment (either alone or with help) or not. The research nurse obtained informed consent from people who were willing and able to participate in the trial. Consenting patients had BP measured electronically three times (a mean of the second and third were calculated) using the IEM stabilograph also used for home monitoring, and daytime ambulatory monitoring using a Spacelabs 902 monitor was initiated unless surgery measured BP was ≤120/60 mmHg or ≥220 mmHg systolic. In these instances the patient would either be advised that they were unlikely to require further treatment to optimise their BP or that their BP was very high, with onward referral to their GP. On return of the ambulatory monitoring equipment, patients whose average daytime ambulatory systolic BP exceeded 130 mmHg were asked to complete a number of questionnaires as baseline measurements including the Hospital Anxiety and Depression Score (HADS) [25], the EQ 5D quality of life measure [26] and the Stroke Impact Scale [27]. Patients’ disability was assessed using the Modified Rankin Scale [28] and with regard to the level of help that may be required from a carer in the use of the equipment. They were randomised after baseline data collection using a remote Internet-based system provided by the Edinburgh Clinical Trials Unit (accessible by telephone if necessary) to telemonitoring or usual care.

All patients were provided with an information pack on lifestyle measures to reduce BP. Intervention patients were taught how to use the equipment. Both the office and daytime ambulatory measurements were reported to the patients’ GP practice. Throughout the trial, patients were reviewed according to clinical need by their usual clinical advisors. Patients were encouraged to contact their doctor in the usual way in emergencies or if concerned. Therapeutic algorithms for both intervention and control groups were provided to practices. These followed existing local guidelines, based on National Institute for Health and Clinical Excellence (NICE)/British Hypertension Society (BHS) guidelines [29,30] (see Additional file 1), but included minimum timelines for escalation of therapy (which had not been needed prior to the introduction of telemonitoring and instant availability of home monitored BP). As with all Lothian guidelines, their use was discretionary.

Patients remained in the trial for 6 months after which the baseline measures were repeated. It was not possible to blind the patients or clinicians to the allocation, potentially introducing bias. However, all trial outcome data collection was undertaken by a second research nurse, blinded to allocation, and patients were requested not to reveal allocation. Patients in the intervention arm were asked to return the telemonitoring equipment to surgery staff when they returned the ambulatory monitor so as not to unblind the research nurse. Use of healthcare resources (number and duration of hospital admissions), practice and out-of-hours consultations, routine reviews for BP, prescriptions for anti-hypertensive drugs and adverse events were extracted from the patients’ primary care record by the research nurse. All adverse events were recorded, specifically noting falls, dizzy spells and postural hypotension. Adverse events were assessed for seriousness by BM, a GP who was blinded to allocation. After the initial data analysis, telemonitoring records were retrieved and analysed for compliance with monitoring.

The sample size was limited to around 50 by the availability of equipment and a target sample size of 56 participants was set, to be randomised in a 3:1 ratio with 42 in the active treatment arm and 14 in the control arm. This ratio was chosen to maximise the number of patients trying telemonitoring whilst still piloting full trial procedures. We were particularly interested in being able to estimate the likely proportion of patients discontinuing telemonitoring. If 6/42 (14.3%) discontinued telemonitoring, this would give a confidence interval (CI) around the estimate of 6.7% to 27.8%. We considered this would provide adequate information to inform the design of the large-scale trial. We also anticipated that stroke patients may be more difficult to recruit than TIA patients. To ensure adequate inclusion of the potentially more disabled stroke patient group, we purposely included 50% stroke patients and 50% TIA patients, stratifying randomisation by stroke/TIA status. Data were analysed using descriptive statistics. Since this pilot was not conducted or powered to detect effects on BP, we did not carry out formal tests of statistical significance.

### Phase 2: Qualitative research

The qualitative part of this mixed methods evaluation had two components because we wanted to explore the experience of those taking part in the pilot trial and the views of a wider group of stroke survivors who may not have expressed interest in the trial because they either could not, or did not want to, monitor their own BP. For the latter, a number of focus groups were recruited from stroke survivors and their carers who were attending Chest Heart and Stroke Scotland (a national charity) stroke support groups. In the focus groups, the equipment was demonstrated and participants were given the opportunity to try fitting the cuff and then asked to discuss whether, if their practice offered it, they would wish to use the home monitoring service. In addition, a purposive sub-sample of patients recruited to the pilot trial were interviewed about their experiences of using the equipment and participating in the trial. The interviews were semi-structured and were undertaken with the help of an interview guide (Additional file 2). Carers were also invited to take part in interviews, if appropriate. A maximum variation sample in relation to patient age, social class, disability and BP control at recruitment was sought for the interviews to elicit the widest range of experience possible. Interviews and focus groups were carried out by PF, a male social scientist, and additional written, informed consent was obtained from all participants. Interviews and focus groups were digitally audio recorded and transcribed in full. Given the communication issues that can follow stroke [31,32], pictorial communication aids (‘talking mats’ [33]) were used to assist expression and comprehension in the focus groups. This approach is described in more detail elsewhere [34]. In order to gain comparative insights, three practice nurses providing the telemonitoring service in this study who had also been involved in our previous BP telemonitoring trial in patients who had not had a stroke or TIA [32] were also interviewed. Interviewing continued until the researcher, in discussion with the wider research team, considered that data saturation was achieved, which, in the context of this study, was considered to have occurred when the researcher was not identifying any new themes and thought this would be unlikely in subsequent interviews.

Data analysis of the interviews and focus groups was thematic. As well as exploring the anticipated themes arising from our previous BP telemonitoring study [35], researchers also specifically searched for emergent, unanticipated themes. A range of strategies was employed to ensure that the analysis was credible and trustworthy. Constant comparison was used to ensure consistency in coding and negative cases were sought for each coding category. Initial coding was carried out by PF. Coding was checked and iteratively refined using paired analysis of transcripts by PF and another researcher. Researcher reflexivity was supported by discussing emerging findings with a wider research group where different explanations were explored and the coding and thematic analysis were reviewed and refined. Following this, the thematic analysis was presented by JH to a discussion group of nine patients, professionals and researchers who had participated in the pilot trial. The presentation introduced the themes and illustrative quotes and the whole data set (all the text associated with each code) was made available to the participants. This discussion, which lasted for 90 min, was moderated by LM.

## Results

### Phase 1: Pilot trial

**Figure 2:**
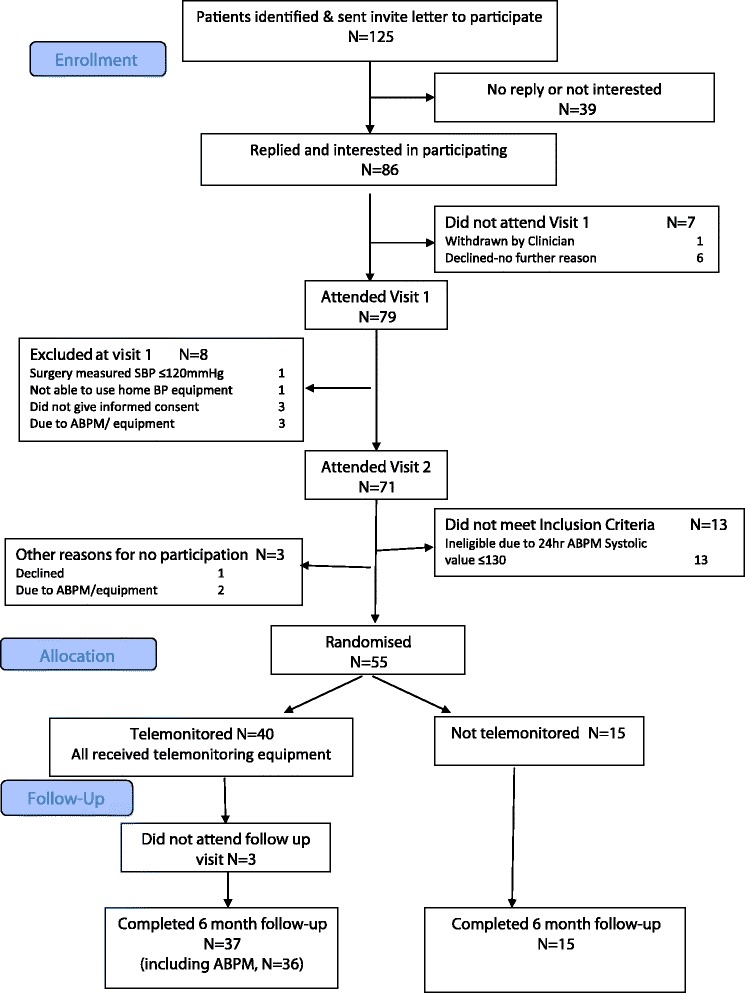
**Patient flow through the study.**

Six general practices participated in the pilot. The subject flow through the pilot trial is shown in Figure 2. Overall, 125 patients (60 stroke patients, 65 TIA patients) were approached and 55 (44%) including 27 (45% of those approached) stroke patients and 28 (43%) TIA patients were randomised. Recruitment stopped at 55 participants because engaging another practice to recruit and manage one additional patient seemed unreasonable. Overall 52 participants (95%) attended the 6-month follow-up appointment but 1 declined the second ABPM measurement resulting in a 93% completion rate for ABPM—the proposed primary outcome measure for a full trial. The participant characteristics are shown in Table 1. Two participants needed help from a carer to use the equipment and they were the same two with moderate/severe disability on the modified Rankin scale. The stroke impact scale (Table 2) confirmed that, as a group, the stroke participants in the pilot trial were not severely disabled. However, at baseline a high proportion of participants [18 (67%) stroke and 22 (79%) TIA patients] had scores on the HADs anxiety subscale of >11, likely to indicate clinically significant anxiety [36].

**Table 1:** **Patient characteristics at baseline**

		**Intervention** ***N*** **= 40**	**Control** ***N*** **= 15**
Male/female (number)	(Total group)	27/13	6/9
	Stroke group	14/6	2/5
	TIA group	13/7	4/4
Age – mean in years (SD)	(Total group)	69.9 (12.6)	73.5 (11.7)
	Stroke	67.8 (14.5)	69.1 (7.9)
	TIA	72.0 (10.2)	77.3 (13.6)
Modified Rankin Scale (stroke patients only) - number			
	No symptoms	7	2
	No significant disability	4	3
	Slight disability	7	1
	Moderate disability	1	1
	Moderately severe disability	1	
Stroke Impact Scale score (stroke patients only) – mean (SD) 0 = no recovery 100 = full recovery	Dimension		
	Physical	78.1 (25.4)	84.8 (19.4)
	Memory	78.9 (23.2)	84.2 (23.7)
	Emotional	78.9 (16.3)	81.0 (11.9)
	Communication	86.3 (19.4)	90.8 (21.3)
	Activities of daily living	82.3 (22.4)	92.9 (5.9)
	Mobility	78.7 (22.0)	85.7 (11.4)
	Hand function	72.5 (30.9)	85.0 (25.7)

**Table 2:** **Outcome measures**

	**Baseline**		**6 months**	
	**Intervention** ***N*** **= 40**	**Control** ***N*** **= 15**	**Intervention N = 37 (36 for ABPM)**	**Control N = 15**
Daytime ABPM systolic mmHg (SD) (proposed primary outcome)	144.1 (10.3)	143.6 (9.6)	133.9 (13.6)	139.8 (15.5)
Daytime ABPM diastolic mmHg (SD)	79.2 (7.8)	77.7 (8.1)	75.2 (7.4)	76.9 (9.6)
Mean HADs Anxiety score (SD)	11.8 (2.7)	12.1(2.4)	12.0 (3.0)	12.9 (1.8)
Mean HADs Depression score (SD)	8.9 (1.7)	9.0 (2.8)	8.7 (1.4)	8.8 (2.1)

Compliance with telemonitoring was good. Of the 40 participants who were telemonitoring, 38 continued to provide readings throughout the 6 months, although one provided fewer than the minimum number of readings requested, giving a rate of 97% full compliance. The outcome measures are shown in Table 2. The mean reduction of 10.1 (SD 15.6) mmHg in systolic ABPM in the monitoring group compared with 3.8 (SD 15.6) mmHg in the control group suggested the potential for a substantial effect from telemonitoring. Overall 15/36 (42%) of the telemonitoring group compared with 2/15 (13%) of the control group reached the target systolic ABPM of < 130 mmHg at follow-up. Mean changes to anxiety and depression scores in the intervention group were minimal. The use of healthcare resources with a comparison to the previous 6 months is shown in Table 3 and changes to anti-hypertensive medication in Table 4. These figures suggest that there was an increase in GP contacts and medication changes in both the intervention and control groups in response to the ABPM results, which were communicated to the practices, and to telemonitoring. There were eight adverse events during the trial period, only two, both non serious, of which may have been BP related, with one of these in each group.

**Table 3:** **Healthcare usage in the 6 months before and after baseline**

	**Total healthcare contacts in 6 months prior to baseline visit**		**Total healthcare contacts in 6 months after baseline visit**	
	**Intervention group** ***N*** **= 37**	**Control group** ***N*** **= 15**	**Intervention group** ***N*** **= 37**	**Control group** ***N*** **= 15**
GP visits/patient	36	33	95	57
Practice nurse visits/patient	64	24	68	36
Phone calls/patient	20	11	48	24
A&E attendances (total)	2	1	2	2
Out of hours (total)	1	1	2	2

**Table 4:** **Anti-hypertensive medication changes during the study**

	**Intervention group** ***N*** **= 37**	**Control group** ***N*** **= 15**
Total number of new drugs prescribed	18	8
Total number of drugs discontinued	5	8
Total number of dosage changes	25	15

### Phase 2: Qualitative research

Sixteen trial participants were interviewed (mean age 66 years, 8 stroke, 8 TIA, 12 male, 4 female) plus 23 patients (7 with their carer) in the three stroke support focus groups (mean patient age 68 years, 11 female, 12 male). The coded data from the transcripts of these interviews were grouped into sub-themes and further grouped into three overarching themes (usual care; trying the new technology; benefits and burdens of telemonitoring) plus a fourth theme on the context of the research study. The first three themes, with illustrative quotes, are presented below. Four professionals were interviewed (three nurses who had also participated in the previous trial of BP telemonitoring, plus a service co-ordinator). The transcripts of interviews with the nurses were read to identify any evidence that they perceived any difference between supporting BP telemonitoring with stroke/TIA patients and other patients. No such evidence was found and no new themes identified compared to the findings of our previous study of BP telemonitoring in non-stroke patients [32]. Therefore, the analysis of the professional interviews is not presented here, but will be included in a wider analysis of professional experience of telemonitoring BP to be published elsewhere.

### Usual care

The participants involved in both the pilot trial and the focus groups were aware that elevated BP is a risk factor for another stroke and were therefore very concerned about it.

*“You’re sent here and you cannae drive your car and you’re just somebody who’s had a stroke and they’re telling you you’re going to have another, and you’re just sitting there wondering if it’s going happen again, hey?”*

*Female, 54, TIA*

However, many were not engaged in their BP management with about half commenting they were never told what their BP reading was when it was checked in the surgery. None of those participating said they owned a BP monitor although two mentioned the possibility.

*“I’ve never seen the results of my blood pressure at any time. Because the nurse takes it, ‘how is it?’ ‘It’s fine’.”*

*Male, focus group (participants only identifiable by sex in focus groups)*

Making appointments and going to the surgery for BP checks was inconvenient and time consuming, especially if appointments were frequent. In addition, patients were aware that surgery BP was not always an accurate indicator.

*“…you have to walk and get an appointment or phone for an appointment and wait 20 hours for it, go to one, go to two, go to three [appointments]…”*

*Male, 62 years, stroke*

*“…where people have white coat syndrome, where as soon as they go to the practice, their blood pressure goes up, you know. And I've always been like that”*

*Male, 64 years, TIA*

### Trying the new technology

Generally, patients participating in the pilot trial found that taking their BP readings and transmitting them was easy and non-intrusive, although a small number felt that they needed some reassurance that they were doing it correctly.

*“I’m quite happy doing it at home, although I think in the very near future I’ll make an appointment with either my GP or the practice nurse, to take a reading on site, just to make sure that it’s actually performing accurately. I don’t believe, for one minute, it’s not, but I think a double check would be in order, because it has, broadly speaking, come down…you’re looking at anything to 15 percent, overall”.*

*Male, 53 years, stroke*

However, when members of the stroke support groups were given the opportunity to try the system, most could not fit the cuff and many said they would not have access to help.

Most people found the system worked smoothly, and any minor technical issues or queries were resolved by the trial manager, who acted as the system manager during the trial. Participants were not always clear about how dialogue with the surgery regarding their measurements should be initiated and, although there was automatic feedback from the system, patients were not always sure that their readings had been reviewed by the practitioners.

*“So the system’s working okay; I don’t know whether they’re reading it, … but the only concern I have now is that the latest one [automatic feedback from system] which I mentioned came in a couple of days ago through the internet, an email expressing some concern. Now, I haven’t had any word from the surgery [name removed] to that effect either, but they may be snowed under or whatever*”.

*Male, 84 years, stroke*

Patients tended to be called in to the surgery if there was concern about their BP and, on some occasions where the nurses monitoring the system were not prescribers, there appeared to be a lack of communication within the healthcare team.

*“Somebody phoned me to say, ‘We’re not happy with your blood pressure reading, you’ve got to go and see your doctor…make an appointment to see your doctor…’ He just said, ‘Oh it’s fine…your blood pressure is fine now’”.*

*Male, 66 years, stroke*

### Benefits and burdens of home telemonitoring

The key benefits reported were a credible estimate of BP, even when it was higher than expected, convenience and reassurance, with the use of the system being preferable to usual care. Patients also thought that their measuring their BP at home released time for the practice.

*“Well, obviously I got a surprise because it's worse than what I already had anticipated it was. It's obviously…I think my BP had been sneaking up a wee bit”.*

*Male, 64 years, TIA*

*“It’s certainly, from my point of view, my selfish point of view, it’s very convenient not to have to make a journey to the doctor’s when it’s only to have BP checked”.*

*Female, 93 years, TIA*

*“…it doesn’t make sense wasting the doctor’s time, wasting my time when I can do it sitting at the table”.*

*Male, 84 years, stroke*

*“It, certainly, takes the worry away, you know, you’re not sitting wondering, you know, I wonder what my BP is, if you’re using that, I’ve never even thought about it”.*

*Male, 70 years, TIA*

The telemonitoring data also supported self-management by some and helped patients to be more engaged in the medical management of their condition.

*“…it went up to 160…And then I was thinking, on the Sunday night I run out of decaffeinated coffee and I started drinking ordinary coffee, and my BP for that week was in the 150 s, right?…I got more decaff and the BP…Aye, it was up here, so it went from 150 down to 130, is it possible?”*

*Male, 80 years, stroke*

The burdens reported by the patients were minimal, the main burden being remembering to take the readings. One person said that abnormal readings could make them anxious.

*“there was a date when I gave some very low readings. That actually worried me more!”*

*Male, 53 years, stroke*

## Discussion

This mixed-methods feasibility study showed that a trial of telemetry supported home BP monitoring for patients who have had stroke/TIA is both feasible and, based on the response rate and qualitative data, would be welcomed by this patient group. Rates of recruitment and retention in the pilot trial were high. The data also suggest that it should be possible to improve BP control in this group. Only a small proportion of those in the pilot trial had significant residual disability, which is consistent with published data on stroke survivors [18], and those who did had assistance to use the equipment. However, the work with the stroke support groups identified some people who would be unable to use the equipment and may not have access to assistance.

The participants in the pilot trial were, on average, older (mean age 71 years) than participants in previous reported trials of BP telemonitoring [13-16]. They also reported more symptoms of anxiety and depression than participants in other trials [16], although there was little change in these measurements associated with the intervention, providing some reassurance that the intervention does not have an adverse effect on mental health. The qualitative data showed that participants were aware of the risk of high BP and further stroke and concerned about it, but about half reported that they had not previously been routinely told their BP measurement when it was checked in the practice. It is therefore unsurprising that many welcomed the opportunity to become more engaged in their BP management and some found home BP monitoring reassuring, as well as being convenient.

This study has demonstrated that improvement in BP control in stroke/TIA survivors is feasible. Based on the results of this pilot, a trial would require 111 subjects per group, assuming a mean systolic ABPM of 134 mmHg (SD 15) with the power to detect a difference of 4 mmHg between groups (based on the outcome of our previous trial [17]) set at 0.9 and α = 0.05. A trial would also be required to estimate the likely impact of BP telemonitoring for this group on health services. As seen previously [17], the introduction of telemonitoring appeared to be associated with an increase in healthcare usage, especially phone calls and medication changes. However, in this feasibility study healthcare usage also increased in the control group where there were, pro rata, more medication changes than in the intervention group. This may have been as a result of the decision to disclose the result of the baseline ABPM for all patients (this was not done in our previous BP telemonitoring trial [17] but was done in this pilot as in the intervening period the NICE guideline 127 [27] had been issued recommending ABPM for diagnosis of hypertension). Alternatively it may have been a chance finding with high consultation rates being a characteristic of this small control group that also had high levels of healthcare consultation prior to the study starting.

## Conclusions

The findings from this study suggest that a full-scale trial is feasible, likely to recruit well and have good rates of compliance and follow-up, and would be required before a definitive statement on the effectiveness of telemonitoring for this group can be made.

### Consents

Written informed consent was obtained for publication of this manuscript and accompanying images by the individuals pictured. A copy of the written consent is available for review by the Editor-in-Chief of this journal.

## Additional files

Additional file 1:
**Rapid Treatment Protocol.** A protocol for managing blood pressure in patients where home monitoring permits rapid escalation of treatment. Additional file 2:
**Interview and Focus Group Guides.**

## Abbreviations

BP: Blood pressure
ABPM: Ambulatory blood pressure monitoring
TIA: Transient ischaemic attack
HADS: Hospital Anxiety and Depression Scale
NICE: National Institute for Health and Clinical Excellence
