# Supplementary material for: Mixed methods feasibility study for a trial of blood pressure telemonitoring for people who have had stroke/transient ischaemic attack (TIA)
Source: Trials. 2015 Mar 25;16:117. doi: 10.1186/s13063-015-0628-y (PMC4404620; doi:10.1186/s13063-015-0628-y)
Supplement: Additional file 1: — Rapid Treatment Protocol. A protocol for managing blood pressure in patients where home monitoring permits rapid escalation of treatment. [file 13063_2015_628_MOESM1_ESM.docx]

Appendix 1: **Rapid Treatment Protocol. A Protocol for managing blood pressure in patients where home monitoring permits rapid escalation of treatment.**

This rapid treatment protocol is based on the Lothian Hypertension guidelines with the addition of timing suggestions and one change. The addition of a thiazide which is an optional first step in the Lothian Hypertension guideline (and also the NICE hypertension guideline) is delayed until step 3 because thiazides take some weeks to show an effect.
