# Supplementary material for: Mixed methods feasibility study for a trial of blood pressure telemonitoring for people who have had stroke/transient ischaemic attack (TIA)
Source: Trials. 2015 Mar 25;16:117. doi: 10.1186/s13063-015-0628-y (PMC4404620; doi:10.1186/s13063-015-0628-y)
Supplement: Additional file 2: — Interview and Focus Group Guides. [file 13063_2015_628_MOESM2_ESM.docx]

**Stroke feasibility trial – focus group schedule**

Preliminaries:

*Completion of consent-to-participation forms.*

*Introductions*

*The qualitative researcher provides a reiteration of the purpose of the focus group to participants.*

*Trial manager’s demonstration of equipment [blood pressure meter, mobile phone, website interface], outline of the telemonitoring activities to be undertaken by patients and practitioners within the trial. [Trial manager departs after demonstration and explanations.]*

*Reminder to participants: we’re not taking blood pressures! No blood pressure readings will be taken as part of involvement in a focus group, but folk are welcome to handle and examine the equipment in order to form first impressions.*

*Reminder of anonymity and confidentiality.*

*Subject/ focus group identifier/ date.*

**1. First impressions: the equipment – blood pressure meter and mobile phone**:

Does the equipment appear to be easy to use?

Any issues in terms of usability?

*Do you think people will be able to use the equipment without assistance?*

*What are the implications if you think assistance is required?*

*What do you think family/ friends will think about telemonitoring?*

**2. First impressions: telemonitoring activities**

Do you think people will find it helpful to be able to see their blood pressure readings throughout their involvement in the research? Why?

People participating in the study will receive text messages by mobile phone about the level of their blood pressure and offering basic advice. Do you think this is a good/ bad thing? Why?

Do you think people will wish to check their readings on the telemonitoring website? Why?

What do you think of the website?

As a result of being telemonitored, people may receive telephone contact with practice staff regarding their blood pressure. Do you think this is a good/ bad thing? Why?

How do you think this type of telemonitoring compares with the usual care you receive from your practice?

*Strengths & weaknesses?*

*Which do you prefer?*

**3. Self management**

Do you think telemonitoring will:

*help people better understands their condition?*

*change how people manage look after themselves?*

*have any impact on people’ quality of life?*

**4. From the demonstration the technology and explanation of what’s involved, what do you think are the best and worst things about being telemonitored?**

**5. Would you telemonitor your blood pressure? Why?**

***What happens next:***

*Transcription, data analysis.*

*Respondents may request access to the focus group transcript and propose amendments*

*re: any errors/ misrepresentations.*

*Respondents will be sent a draft copy of the research report for comments/ approval.*

**Stroke feasibility trial – patient interview schedule (midpoint interviews)**

*Subject/ trial number/ initials /region / date*

*Statement of anonymity.*

*Would like to talk about:*

- *how you got involved in the research project*
- *your experience of involvement in the research project*
- *what you think about the equipment and your experience of using it*
- *your views about telemonitoring*
- *Anything else you think it may be helpful for us to know*

**1. Getting involved in the research**

How did it happen?

What were your reasons for getting involved?

Has information from the research team been clear to understand?

Did you read the research leaflets?

*Did they make sense to you?*

How did you find attending the research appointments at practice?

Did you receive training in using the equipment?

*If so, what did you think of the training you’ve received?*

How did you find taking initial blood pressure over the 24hour period using the special device given to you at the beginning of your involvement in the study?

**2. Using the equipment**:

Tell me about your telemonitoring routine.

*What is it you do?*

*How often? When do you take your readings? What do you do during holidays?*

*Does the telemonitoring routine (taking readings) impact on your day-to-day life*? If so, how?

How do you find using the technology?

Any technical difficulties with the equipment?

*Has it been explained to you what to do in case of technical problems?*

*Have you had technical assistance for any problems?*

*If so, what did you think of the support received to address technical difficulties?*

**3. Undertaking telemonitoring:**

Tell me what happens after you send your readings?

Have you had contact from healthcare professionals as a result of being telemonitored? If so, what about?

Have you received any text messages by mobile phone about the level of your blood pressure. How did you feel about getting these texts? What did you do as a result of receiving the texts? Do you think getting such texts this is a good/ bad thing? Why?

Do you check your readings on the website? If so, what do you think of the website?

What do you think of the service you have received as a result of being telemonitored?

*How does it compare to the care you usually receive? Which do you prefer?*

What do you think healthcare professionals involved in your care think about telemonitoring?

**4. Telemonitoring and looking after yourself**

Do you find it helpful to be able to see your blood pressure readings?

Has telemonitoring:

*helped you better understand your condition?*

*affected how you feel about your condition and/ or care you receive?*

*led to any changes in how you look after yourself?*

*had any impact on your quality of life?*

**5. Possible involvement of family & friends**

Have family and friends seen the equipment? If so, what have they said about it?

Do you think it has affected what they know about your condition?

Are family/ friends involved in helping you with telemonitoring? If so, how?

**6. What are the best and worst things about being telemonitored?**

**7. The trial of this technology is for a fixed period. However, in an ideal world where the technology is considered useful and it was funded and provided by NHS Lothian, and if given the choice, would you continue telemonitoring after your involvement in the research is over?**

***What happens next:***

*Transcription, data analysis.*

*Respondents may request access to their own transcript and propose amendments*

*re: any errors/ misrepresentations.*

*Respondents will be sent a draft copy of the research report for comments/ approval.*
